# Understanding How Regional Quota Medical Students in Okinawa Develop Their Specialty Preferences: A Qualitative Study of Career Development and Professional Identity Formation

**DOI:** 10.7759/cureus.98550

**Published:** 2025-12-05

**Authors:** Hirotake Machida, Yuiko Hiyajo, Richi Kakazu, Miyu Yara, Kiyoshi Kinjo, Ryuichi Ohta

**Affiliations:** 1 Family Medicine, Faculty of Medicine, University of the Ryukyus, Okinawa, JPN; 2 Medical Education, Faculty of Medicine, University of the Ryukyus, Okinawa, JPN; 3 Community Care, Unnan City Hospital, Unnan, JPN

**Keywords:** career choice, chiikiwaku, general medicine, medical education, physician distribution, professional identity, qualitative research, regional quota system, rural health services

## Abstract

Introduction

Japan’s Regional Quota System (ChiikiWaku) was established to address physician shortages in rural areas by supporting medical students with scholarships in exchange for service commitments. However, the specialty choices of quota students sometimes diverge from the regional healthcare needs. This study explored how specialty preferences change during medical education among regional quota students at the University of the Ryukyus in the Okinawa Prefecture, a region facing persistent healthcare disparities.

Methods

A qualitative study using semi-structured interviews was conducted with regional quota students across different academic years at the University of the Ryukyus. Semi-structured interviews were selected for their flexibility and ability to capture in-depth experiences. Participants were purposively sampled to capture diverse perspectives. The interviews were audio-recorded, transcribed verbatim, and analyzed thematically by a multidisciplinary research team, including quota and non-quota students, to enhance reflexivity and credibility.

Results

Thematic analysis identified four central themes: (1) developing interests in various specialties through studying medicine, (2) hope and anxiety toward diverse futures, (3) dilemma between specialty limitations and the desire for career diversity, and (4) ambivalence toward general practice and its professional identity. The students' specialty preferences evolved through educational exposure, personal experiences, and negotiation between aspirations and systemic obligations. General practice was seen with both appreciation and uncertainty, reflecting the complex interplay between personal and policy-driven career trajectories.

Conclusion

Regional quota students’ specialty choices are dynamic and influenced by multiple factors during their medical education. Tailored support, including mentorship and early exposure to community-relevant specialties, may help better align the evolving aspirations of the students with the healthcare needs of underserved regions.

## Introduction

In the recent decades, Japan has implemented various strategies to address physician shortages in the rural and underserved regions [1]. Among these initiatives, the regional quota system (ChiikiWaku) in medical schools has emerged as a pivotal policy tool designed to reinforce healthcare delivery in such areas [2]. This system enables students to enter medical school with the support of scholarships or tuition waivers because they will serve in specific regions after graduation [3]. While this system plays a crucial role in securing future healthcare providers for medically underserved areas, concerns have arisen regarding the actual post-graduation career paths of students admitted under this framework [4]. Despite the system’s intention to direct graduates toward community-based medical practice, evidence suggests considerable variation in their specialty and practice location choices [5]. Understanding this variability requires examining not only workforce outcomes but also the mechanisms shaping student decisions. Established frameworks, such as the Theory of Career Choice, models of professional identity formation, and rural workforce pipeline theory, suggest that career trajectories are influenced by evolving values, perceptions of autonomy, and experiential learning rather than policy requirements alone [5]. A qualitative exploration of the students’ lived experiences can therefore clarify how these mechanisms operate within the regional quota context, offering insight into why intended rural practice pathways may diverge from policy expectations [6-8]. These insights are essential for informing policy planning and aligning physician distribution with regional healthcare needs.

Each region in Japan faces distinct medical challenges, and the specialties most in demand can differ markedly. For instance, some areas struggle to secure pediatricians, obstetricians-gynecologists, and emergency medicine specialists [6]. In the Okinawa Prefecture, where geographic and socioeconomic factors contribute to persistent health disparities, ensuring a stable physician supply in key specialties remains a critical concern [7,8]. Against this backdrop, the specialty preferences and eventual career choices of regional quota students at the University of the Ryukyus School of Medicine carry substantial implications for the sustainability of local healthcare services [9,10]. Understanding the trajectories of these students, including how their preferences evolve during medical school, is essential for designing effective workforce policies and improving healthcare equity in remote and underserved communities.

One notable issue that has garnered attention is the misalignment between the specialties regional quota students intend to pursue and the actual needs of the communities they are expected to serve. National workforce reports and previous analyses have documented persistent shortages in core community-based specialties, such as general medicine, pediatrics, obstetrics-gynecology, and emergency care, despite an increasing number of regional quota entrants [11]. Recent national data also show that a substantial proportion of ChiikiWaku graduates select hospital-based subspecialties rather than primary care or rural-relevant fields [6,9,10], indicating that this misalignment is not merely anecdotal but quantitatively observed. This gap may stem from multiple factors, including limited opportunities for students to engage with regional healthcare settings during training, a lack of mentorship in community-relevant specialties, and personal aspirations that diverge from policy expectations [12].

Such discrepancies threaten the effectiveness of the regional quota system, potentially undermining the goal of alleviating physician shortages in critical specialties. To strengthen policy relevance, it is crucial not only to quantify the extent of this mismatch but also to understand the mechanisms that shape the evolving specialty preferences of the students. A qualitative approach allows exploration of these mechanisms, such as identity formation, perceived autonomy, value shifts, and clinical exposure, which cannot be fully captured through quantitative workforce data alone. Addressing this issue requires not only identifying the extent of this mismatch but also understanding the underlying motivations and influences that shape the career choices of students.

This study explores the perceptions of the regional quota students, of how their specialty preferences are shaped during their medical education, and identifies the factors that contribute to these shifts in perspective. Focusing on students in the Okinawa Prefecture, this research seeks to provide insights into the broader challenges of aligning medical education with regional healthcare needs. The findings of this study are expected to contribute to the development of more responsive medical education strategies, inform local health policy, and enhance support systems for regional quota students, ultimately strengthening the regional quota system's capacity to achieve its intended outcomes in community-based medical care. Rather than documenting actual longitudinal changes, this study examines how students interpret and make meaning of the influences on their evolving career orientations.

## Materials and methods

Setting

This study was conducted at the Faculty of Medicine, University of the Ryukyus, in the Okinawa Prefecture, Japan. The university is situated in a geographically and culturally distinct region that faces significant challenges in healthcare access, including persistent physician shortages, limited specialist availability on remote islands, and marked rural-urban disparities in service provision, making it a relevant setting for examining the education and development of medical students admitted through the regional quota (ChiikiWaku) system for the solution of healthcare disparities. Each year, the faculty admits about 110 students, of whom approximately 17 are enrolled under the ChiikiWaku program (14 regional quota and three remote/island quota students). These students receive loan-based scholarships from the prefectural government, which covers tuition and living expenses. They are obligated to serve as physicians for nine to 14 years after graduation, including four years of service in the remote or northern areas of the prefecture [10]. The study was conducted in collaboration with faculty members and administrative personnel from the university and related institutions, taking into account the local healthcare context and the background of the regional quota program.

Participants

Participants were medical students enrolled through the regional quota system at the University of the Ryukyus. Inclusion criteria encompassed students from all academic years to capture various educational experiences and perspectives. A purposive sampling strategy was employed to ensure diversity across gender, year of study, and regional background. Diversity was operationalized by seeking representation from male and female students across mainland Okinawa and remote island regions. Moreover, to explore changes in student attitudes and identity formation, students were selected to represent different stages of their medical education, and included first-year to fourth-year students. This theoretically informed sampling strategy allowed the research team to capture a wide range of interpretive perspectives rather than a statistically representative cohort. This approach allowed the research team to capture longitudinal perspectives and trace potential shifts in specialty preferences and professional identity.

Data collection

Data were collected through semi-structured one-on-one interviews, conducted in person or via secure online platforms, depending on participants' preferences and availability. Each interview lasted approximately 45 to 60 minutes and was audio-recorded with the participant’s consent. The interview guide guided the interviews. It consisted of the following key questions: “Did your future specialty choice change after admission to medical school?” “What factors influenced your thinking about specialty choice?” “How do you perceive the expectations and obligations of the regional quota system?” “What experiences during medical school shaped your interest or hesitation toward specific specialties?” and “What challenges or concerns do you feel when imagining your future specialty?” These guiding questions were used flexibly to encourage participants to elaborate on their experiences and perspectives. Interview guides were designed to elicit detailed narratives about participants’ motivations, expectations, challenges, and perceived educational support related to their status as ChiikiWaku students. Interviewers were trained in qualitative interviewing techniques to ensure consistent and in-depth data collection. Given that the interviewers were peers of some participants, we incorporated explicit reflexivity practices to address potential positionality and power-dynamic concerns. Interviewers maintained reflexive journals to document assumptions and interpersonal influences, clarified their role as researchers rather than evaluators or seniors, and emphasized voluntary participation and confidentiality to reduce social desirability pressures. To mitigate bias during analysis, initial coding was cross-checked by researchers not involved in the interviews, and emerging themes were reviewed collaboratively to ensure interpretations were not shaped solely by peer relationships. These procedures, together with triangulation through multiple analysts, served as analytic safeguards to enhance credibility and trustworthiness.

Data analysis

Interviews were transcribed verbatim in Japanese. A thematic analysis approach was employed to analyze the qualitative data [13]. We conducted an inductive thematic analysis following Braun and Clarke’s six-phase framework, allowing themes to emerge from the data rather than predefined categories. The two researchers (HM and RO) independently read and coded the transcripts to identify initial patterns and concepts. Codes were first generated line by line, recorded in analytic memos, and organized into an evolving codebook. An audit trail documenting coding decisions, code merges, and revisions was maintained throughout the analysis. These codes were then compared, discussed, and refined through a consensus-building process. Coder disagreements were resolved through iterative discussion, and when consensus could not be reached, a third researcher (YH) adjudicated. Qualitative data analysis software (NVivo 12, QSR International, US) was used to manage transcripts, organize codes, and support transparent tracking of analytic changes. Central themes were extracted by grouping codes into broader categories representing key aspects of the students’ experiences and perceptions. The thematic framework was iteratively refined until thematic saturation was achieved. A comprehensive discussion was conducted to enhance the trustworthiness and credibility of the analysis among the researchers (HM, YH, RK, MY, and RO). Preliminary findings were shared with KK, who provided feedback on whether the results accurately reflected his view as a medical teacher. Discrepancies were discussed and incorporated into the final thematic structure.

Reflexivity

This study was conducted collaboratively through interactions between the researchers and participants. The research team had diverse expertise and perspectives on rural community care and medical education. RK, HM, MY, and YH were medical students at the University of the Ryukyus. RK, MY, and YH were students from the regional quota. HM was a student not from the regional quota. Four researchers participated in the semi-structured interviews. RO is a family physician and public health professional who graduated with a master's degree in public health and family medicine and has experience conducting research on rural community healthcare. KK was a professor in the Faculty of Medicine at the University of the Ryukyus and managed the education of ChiikiWaku. To minimize bias, each idea related to the conditions of motives among medical students from the regional quota was discussed by analyzing the research content of the individual data analyses. Alternative viewpoints were explored during the data interpretation stage.

Ethical considerations

Ethical approval for this study was obtained from the Clinical Ethics Committee of the Unnan City Hospital (approval code: 20230039). All participants received a detailed explanation of the study's purpose, procedures, confidentiality safeguards, and their right to withdraw without penalty. Written informed consent was obtained from all participants before the interviews. All data were anonymized, securely stored, and used solely for this research.

## Results

The study participants consisted of 12 students: two were in their fourth year, six in their third year, one in the second year, and three in the first year. The gender distribution was three males and nine females. Regarding their background, nine students were from the regional quota, with origins on the main island of Okinawa. Through thematic analysis of semi-structured interviews with regional quota students at the University of the Ryukyus, four central themes emerged on how students interpret and make sense of influences on their specialty preferences, rather than documenting actual changes over time. These themes captured the participants’ perceived trajectories and retrospective reconstructions, of how their values and career orientations evolved, as understood within an interpretivist framework. They illustrate the students' evolving sense of professional identity, emotional tensions between systemic obligation and personal aspiration, and moments of development through medical learning (Table 1).

**Table 1:** Summary of themes and associated concepts identified through the thematic analysis This table provides a structured overview of each theme and its underlying concepts.

Themes	Concepts
Developing interests in various specialties through studying medicine	Learning connected to daily life, interest in basic science, curiosity across specialties
Hope and anxiety toward diverse futures	Openness to exploration, interest in local and diverse practice, communication concerns
Dilemma between specialty limitations and the desire for career diversity	Lack of clarity, acceptance of restrictions, career choice shaped by community need
Ambivalence toward general practice and its professional identity	Unclear identity of general practice, positive emotional image of home care, desire for variety in practice

**Figure 1:**
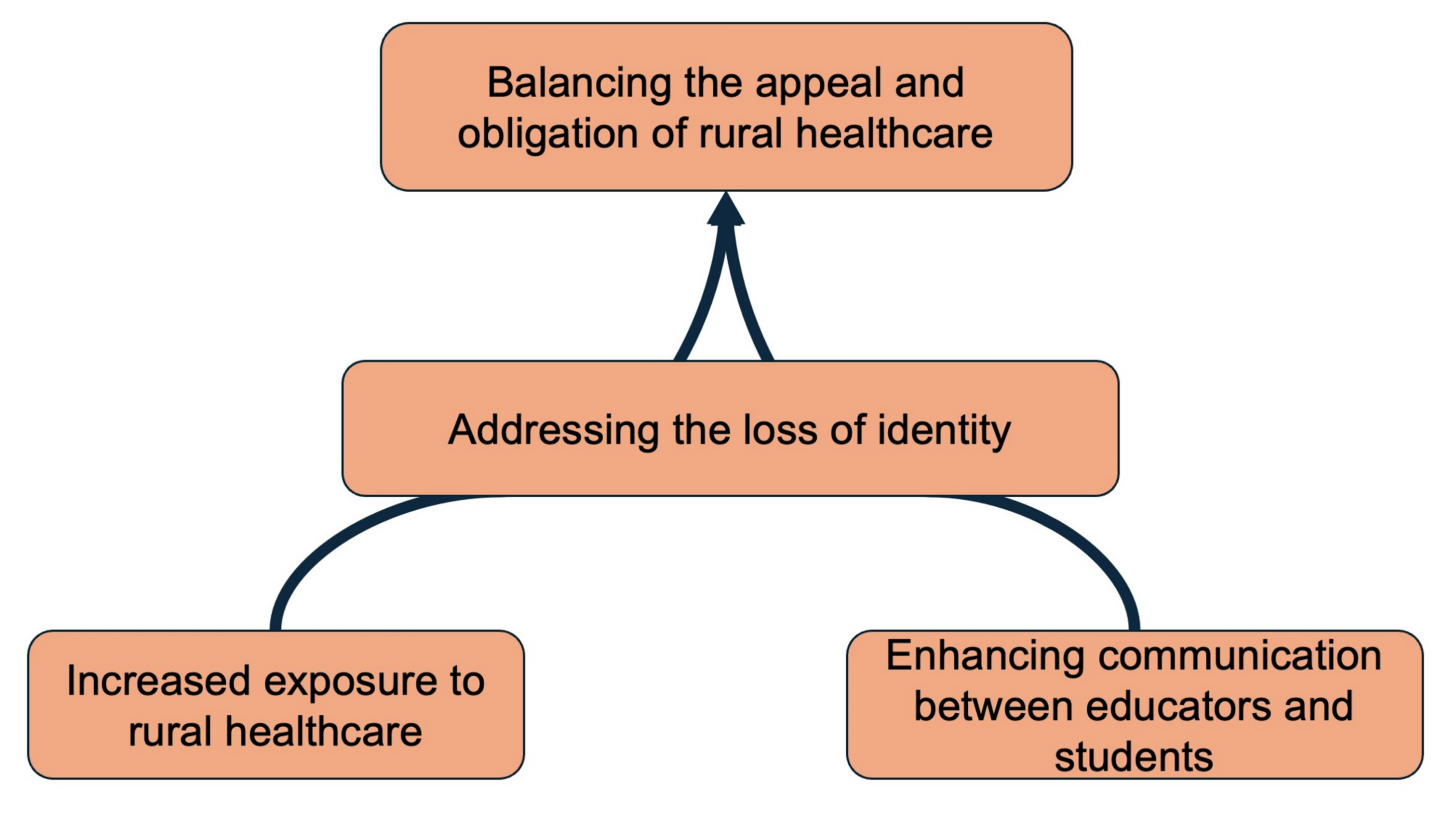
Conceptual model illustrating the relationships and developmental flow among the four themes This figure visually represents how the themes interact and influence the formation of specialty preferences. Image credit: Created by Ohta R using Microsoft PowerPoint (Microsoft Corporation, Redmond, WA, USA).

The conceptual figure is shown in Figure 1.

Developing interests in various specialties through studying medicine

Many students described how the enjoyment of learning medicine sparked an early interest in specific specialties. These interests often stemmed from moments when medical knowledge intersected with their personal lives or intellectual curiosity. Interviewee number two recalled a time when studying dermatology overlapped with individual experience as follows: “While studying dermatology, I happened to be having skin issues myself. It was fascinating to apply what I was learning directly to my life.” 

Others found excitement in the invisible mechanics of the human body through basic science. For example, interviewee number four expressed: “I think many students are interested in basic sciences. Something is intriguing about thinking through mechanisms that you can’t see.” 

Some participants were generally curious about various specialties and diagnostics, preferring to explore options before making firm commitments. This exploratory stance, found in students like interviewee number six, reflected an eagerness to experience diverse clinical areas before deciding on a path.

Hope and anxiety toward diverse futures

A common thread in many narratives was the coexistence of hopeful curiosity and subtle anxiety about the future. Students often expressed a desire to keep their options open, hoping that experiences in medical school would help them clarify their direction. Interviewee number one reflected: “I want to try living life in a new way-not just something linear or predetermined.” Interviewee number six echoed this openness, especially concerning the quota system’s requirement to declare future preferences annually: “Because we have to submit our preferences every year, it’s a good opportunity to re-think what direction I want to go in.” However, underlying these expressions of openness were moments of vulnerability. Interviewee number two shared their concern about interpersonal demands in clinical practice: “I’m worried that I may not be good at communicating with patients in stressful situations. That makes me feel nervous.” Such reflections show that while students value diverse experiences, uncertainty and self-doubt can complicate their career planning process.

Dilemma between specialty limitations and the desire for career diversity

Students acknowledged the constraints of the regional quota system with varying degrees of acceptance and internal conflict. Some expressed quiet resignation, feeling that their preferred specialty paths might not be fully compatible with system expectations. Interviewee number 11, for instance, remarked: “To be honest, I’ll prioritize the quota obligations. It’s something I’ve accepted-it’s inevitable.” Later, the same student added: “If I have to change my plans, I guess it can’t be helped. Honestly, it just feels like something I have to accept.” These statements reveal a subtle but essential internal negotiation. While these students respect the social purpose of the system, they also feel that it requires sacrificing parts of their ideal career pathways.

Some students, like interviewee number six, tried to reframe this constraint through a social lens: “Even if it’s not my top choice, if the region needs it, I feel I should choose that path.” This pragmatic perspective reflects empathy and compromise, balancing self-identity with societal obligation.

Ambivalence toward general practice and its professional identity

General practice, a commonly expected pathway for quota students, elicited mixed emotions. Some students viewed it as meaningful and community-focused, while others struggled with its ambiguous professional image. Interviewee number six stated frankly: “I still don’t have confidence in being a generalist. Compared to other specialties, it feels vaguer and more undefined.” In contrast, interviewee number three shared a more emotionally grounded impression of generalist work in home care: “There’s something warm about home visits. It feels more personal and human.” These views suggest that general practice evokes both uncertainty and interest. Students appreciated its diversity and flexibility, but some lacked confidence in their readiness to embody its multifaceted demands.

## Discussion

Summary of the research

This qualitative study explored how regional quota medical students at the University of the Ryukyus in the Okinawa Prefecture perceived and reflected on the development of their specialty preferences during their education. Rather than tracing actual changes over time, the study revealed how the students’ narratives reflected evolving understanding and tension surrounding their choice of specialty. Four central themes emerged: discovering specialty interests through the joy of learning medicine, hope and anxiety toward diverse futures, dilemmas between specialty limitations and the desire for career diversity, and ambivalence toward general practice and its professional identity.

The students’ specialty choices evolved through personal experiences, educational exposure, and ongoing negotiation between their aspirations and systemic expectations. The ambiguity surrounding general practice and the perceived constraints of regional quota obligations contributed to their perceptions of uncertainty about their future careers. These interpretive patterns align with the uncertainty management theory and models of professional identity formation, suggesting that students navigate ambiguous expectations by oscillating between personal aspirations and perceived systemic requirements.

Comparisons with other studies

The findings of this study are consistent with previous research on regional quota systems and medical education in rural areas. Earlier studies in Japan and other countries have indicated that while regional admission policies successfully channel students toward underserved regions, the alignment between their specialty preferences and regional healthcare needs often remains imperfect [5,14-16]. Similar to the current findings, prior research has reported that students initially exhibit openness to a wide range of specialties but later encounter tension between personal interests and societal obligations [1,17,18]. Our results deepen this understanding by showing how students interpret these tensions through processes of identity negotiation, rather than viewing them as purely structural mismatches. The ambivalence toward general practice observed in this study mirrors findings from studies in Australia and Canada, where students admitted through rural pipelines expressed both appreciation for and hesitancy toward rural generalist careers due to unclear professional identity and role expectations [19-22]. However, our findings extend this literature by highlighting the emotional and interpretive work students perform when confronting such ambiguity, suggesting that uncertainty arises not only from limited knowledge of general practice but also from perceived incongruence between their emerging identities and externally imposed expectations. Additionally, global studies on medical student career development emphasize the importance of joyful, meaningful learning experiences in shaping early specialty interests, reinforcing the critical role of diverse and engaging educational experiences in fostering specialty commitment [23-25]. In line with these studies, the accounts of our participants illustrate how emotionally resonant learning moments contribute to the perceived career fit, offering a nuanced view of how experiential meaning-making, rather than exposure alone, supports specialty preference formation within the regional quota context.

Strengths of the study

One of the significant strengths of this study lies in its in-depth exploration of the students’ evolving perceptions through a qualitative approach. The study captured developmental perspectives on specialty decision-making by including students from different academic years and stages of professional identity formation. The study also benefited from reflexivity, with multiple researchers, including regional quota students, participating in the coding and analysis process, thereby enriching the interpretive depth and credibility of the findings. Furthermore, the specific focus on the Okinawa Prefecture, a region with significant healthcare disparities, adds valuable context-specific insights that can inform both local and national medical education policies.

Limitations

Several limitations should be acknowledged. First, the study was conducted at a single institution, which may limit the transferability of the findings to other medical schools with different educational cultures or regional demands. Second, while purposive sampling aimed for diversity, the sample size remains relatively small, and selection bias cannot be ruled out, as participants who were more engaged or reflective about their career choices may have been more likely to participate. In a thematic analysis, adequacy is determined not by sample size but by the richness and relevance of the data; in this study, the interviews provided sufficiently detailed and nuanced accounts to support meaningful pattern development. Consistent with the concept of information power, the focused research aim, the relative homogeneity of the participant group, and the depth of participants’ narratives contributed to analytic sufficiency. Third, the study relied on self-reported data, which are subject to recall bias and social desirability bias. Although peer interviewers may have influenced participants’ openness, the diversity of viewpoints across levels of years, genders, and regional origins suggests that the composition of participants did not disproportionately skew the findings but rather enabled the identification of recurring interpretive patterns. Lastly, the longitudinal aspect was reconstructed through retrospective narratives rather than prospective follow-up, which may affect the accuracy of the recollections of the participants about their changing specialty preferences [26].

## Conclusions

This study highlights how regional quota students at the University of the Ryukyus navigate the complex interplay between personal aspirations, policy expectations, and the meanings they attach to their clinical experiences. Students described both joyful discovery through medical learning and emotional tensions arising from structural obligations and ambiguity surrounding general practice. Their narratives illustrate that specialty decision-making is shaped not only by exposure but also by evolving professional identities and the interpretive processes through which students assess career fit. These findings suggest that early and meaningful engagement with community-relevant specialties, along with clearer role modeling and mentorship in general practice, can help students navigate uncertainty. Supportive learning environments that acknowledge policy-related pressures may further ease identity conflicts and promote more confident career trajectories. Overall, the study proposes a conceptual model in which policy constraints, identity formation, and experiential learning together shape specialty preferences, offering guidance for educational strategies that better align student development with rural healthcare needs.
